# Evaluation and validation of 3D-printed anatomical urinary system model and virtual reality RIRS simulators in RIRS training: a comparative study

**DOI:** 10.55730/1300-0144.6022

**Published:** 2025-06-05

**Authors:** Mehmet EZER, Tahsin Batuhan AYDOĞAN, Lazaros TZELVES, Andreas SKOLARIKOS, Mehmet USLU, Kemal SARICA, Emre HURİ

**Affiliations:** 1 Department of Urology, School of Medicine, Kafhas University, Kars, Turkiye; 2 Department of Urology, Kartal Dr. Lütfi Kırdar City Hospital, İstanbul, Turkiye; 3 Department of Uro-Oncology, University College of London Hospitals, UCLH, London, United Kingdom; 4 Second Department of Urology, Sismanoglio Hospital, Athens, Greece; 5 Department of Urology, Sancaktepe Şehit Prof. Dr. İlhan Varank Training and Research Hospital, İstanbul, Turkiye; 6 Department of Urology, Biruni University Medical School, İstanbul, Turkiye; 7 Department of Urology, School of Medicine, Hacettepe University, Ankara, Turkiye

**Keywords:** RIRS, 3D printing, simulation, virtual reality, surgical training

## Abstract

**Background/aim:**

This study evaluates the use of a 3D-printed anatomical urinary system model (3D-AUSM) and a Virtual Reality RIRS Simulator (VRRS), for training in RIRS, based on real user feedback.

**Materials and methods:**

The 3D-AUSM was created using cadaver CT and MRI scans, and the data was transferred to a VR environment for simulation. A total of 43 inexperienced urology trainees participated in the theoretical phase of the RIRS training program. Of these, 32 trainees (Group T) who passed a proficiency exam proceeded to the hands-on training phase with the 3D-AUSM and VRRS models. Additionally, 17 experienced surgeons (Group S) were included in the study for validation purposes. Skill scores and procedure times were recorded for both groups, and participants completed surveys to evaluate content, face, and construct validation of the models.

**Results:**

Group S completed the procedures faster and achieved higher skill scores than Group T in both models. Group T, however, performed better with VRRS compared to 3D-AUSM. The most challenging steps for both groups were “exposing the intrarenal collecting system” and “relocating the stone”. Both groups rated the models highly for content and face validation, though experienced surgeons gave lower overall satisfaction scores to VRRS compared to 3D-AUSM.

**Conclusion:**

3D-printed models and VR simulators are safe, cost-effective tools for developing essential surgical skills. While 3D-AUSM provides realistic anatomical feedback, VRRS offers unlimited practice opportunities. Both models are valuable in surgical education, promoting standardized, effective training.

## 1. Introduction

Retrograde intrarenal surgery (RIRS) is a widely used treatment option for kidney stones, particularly with the effective use of the Holmium-YAG laser. While it has predominantly been used for stones smaller than 2 cm, recent studies suggest that it can also be successfully applied to manage stones larger than 2 cm with low complication rates and high success rates [1]. RIRS, being minimally invasive, offers several advantages over traditional methods such as percutaneous nephrolithotomy, particularly for stones in difficult-to-reach areas of the kidney, making it an increasingly favored technique [2].

Despite the seemingly practical nature of RIRS, the procedure involves numerous complex steps and requires precise anatomical orientation within the renal collecting system. Additionally, the lack of tactile feedback when using a flexible ureteroscope and the need for multiple devices make the procedure particularly challenging for beginners [3]. The steep learning curve of RIRS, owing to the lack of haptic feedback and reliance on precise visual cues, further emphasizes the necessity of effective training modalities to ensure competency before clinical application [4]. As a result, a structured, standardized training phase is essential to ensure safe and accurate application in clinical practice.

Given the ethical and medicolegal concerns surrounding patient safety, it is evident that new and inexperienced surgeons cannot develop these critical skills solely through direct patient applications. The importance of gaining these fundamental skills in a risk-free, simulated environment highlights the need for developing innovative, practical training models [5]. Unlike traditional surgical training, these models offer a repeatable, risk-free environment where complex procedures can be practiced, allowing trainees to develop essential skills without compromising patient safety.

In recent years, virtual reality simulators and 3D-printed models, designed using computer-aided technologies, have been widely adopted in various fields such as engineering, training laboratories, and aviation [6]. Medicine has also seen significant growth in the use of these technologies over the past few decades [7]. Although various training models have been introduced, comprehensive studies evaluating the effectiveness of these models in RIRS training, particularly in terms of user experience and skill acquisition, remain limited [8–10].

This study aims to evaluate the effectiveness of two different training modalities—the 3D-printed anatomical urinary system model (3D-AUSM) and the Virtual Reality RIRS Simulator (VRRS)-developed using cadaver-based computerized tomography (CT) images in RIRS training, based on user experiences.

## 2. Materials and methods

### 2.1. 3D modelling and preparation of educational materials

**Figure 1:**
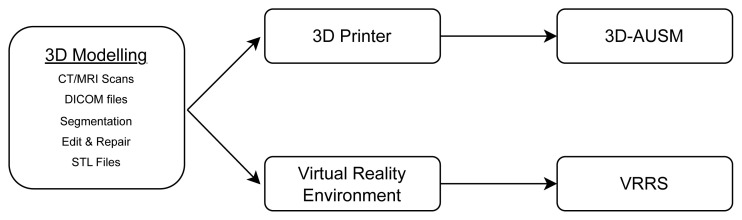
Overview of the 3D modeling process and preparation of educational materials. The 3D modeling process begins with CT/MRI scans and DICOM files, followed by segmentation and editing to produce stereolithography (STL) files. These STL files are then used to create 3D-printed anatomical models (3D-AUSM) or transferred to a virtual reality environment (VRRS) for simulation-based training. (CT: Computed Tomography, MRI: Magnetic Resonance Imaging, DICOM: Digital Imaging and Communications in Medicine, STL: Stereolithography, 3D-AUSM: 3D-Printed Anatomical Urinary System Model, VRRS: Virtual Reality RIRS Simulator)

The 3D kidney modeling process using cadaver images, as shown in Figure 1, was the first step of this study. For this, Digital Imaging and Communication in Medicine (DICOM) files of a real cadaver, with radiological imaging permission obtained through a protocol with the Anatomy department (project approval: 07/12/2020, 2020-1-TR01-KA203-093898). The anatomical 3D models were created using Materialise Interactive Medical Image Control System (Mimics) software (Materialise NV) by the project team. Further revisions and repairs were made using 3DS MAX (Autodesk) and Zbrush (Pixologic) 3D model editing software. Textures were applied using Photoshop (Adobe), and polygonal mesh (stereolithography, .stl) files were generated for 3D printing to produce realistic anatomical structures.

**Figure 2:**
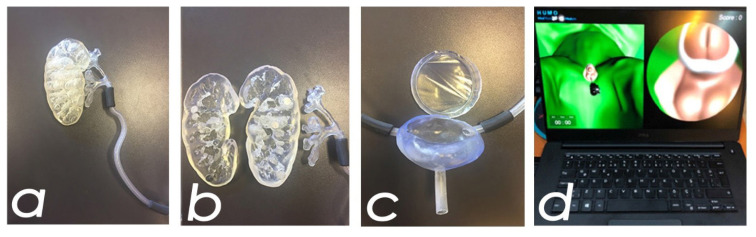
Components of the 3D Anatomical Urinary System Model: (a) external view of the kidney model, (b) cross-sectional view of the kidney model showing inner structures, (c) bladder model, and (d) a screenshot of the VR RIRS simulator. (3D: three-dimensional, VR: virtual reality, RIRS: retrograde intrarenal surgery).

The 3D anatomical urinary system model consisted of three parts: the kidney, ureter, and bladder. The kidney model was produced over 16 h using stereolithography (SLA) technology, with a production resolution of 0.025 mm, using resin/hard material. The ureter model was created in 8 h using the same technology, material, and resolution. The bladder model was produced in 10 h with a resolution of 0.1 mm, using the same technology and material. After completing the production and postprocessing steps, the kidney, ureter, and bladder models were combined to form the 3D-AUSM, as shown in Figure 2a-d.

The created 3D model data was then transferred to a virtual reality (VR) environment using Unity Real-Time Development Platform (Unity Technologies), where it was transformed into a game-based simulation for training purposes (VRRS), as shown in Figure 2.

### 2.2. Study protocol

**Figure 3:**
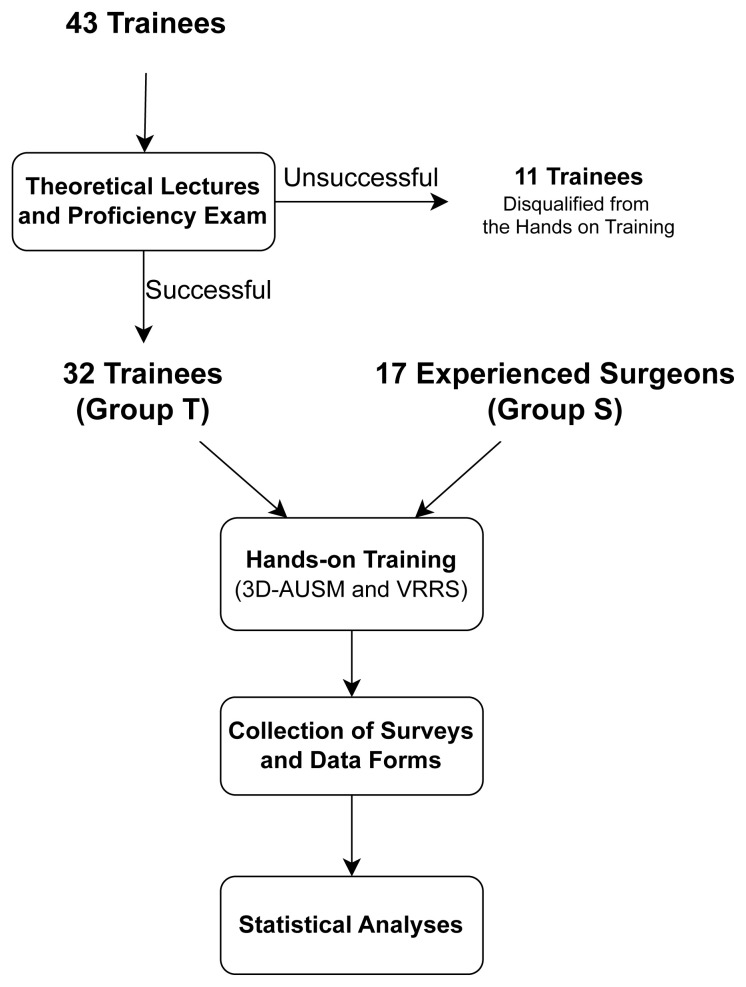
Flowchart illustrating the study protocol. A total of 43 trainees participated in the theoretical lectures and proficiency exam. Of these, 32 successful trainees proceeded to the hands-on training (Group T), while 11 trainees who did not pass the exam were disqualified. Additionally, 17 experienced surgeons (Group S) participated in the hands-on training for validation purposes. The study proceeded with hands-on training using 3D-AUSM and VRRS, followed by the collection of surveys and data forms for statistical analysis. (3D-AUSM: 3D-Printed Anatomical Urinary System Model, VRRS: Virtual Reality RIRS Simulator, RIRS: Retrograde Intrarenal Surgery).

The training protocol was divided into four sessions, as outlined in the flowchart shown in Figure 3.

**Session I - Theoretical lectures:** Within the scope of the project, a meeting was organized to evaluate the use of these developed models in RIRS training. The inclusion criteria required participants to be urology residents, with no prior RIRS experience (<10 RIRS cases), and an interest in learning new training modalities and technologies. A total of 43 trainees attended the RIRS training sessions held within the scope of the project. All participants received two hours of theoretical lectures from mentors who had performed more than 100 RIRS cases, using surgical videos from actual operations. The lectures covered urinary system anatomy, RIRS indications, procedures, complications, and surgical instruments.

**Session II - Proficiency exam:** After the theoretical lectures, trainees took a 20-question proficiency exam, with each question worth 5 points. Trainees who scored 90 or above were allowed to proceed to hands-on training with the 3D-AUSM and VRRS. Of the 43 participants, 32 passed the exam and continued to the hands-on training phase.

**Session III – Hands-on training:** The 32 trainees who passed the exam formed Group T (Trainees) in the hands-on training phase. A second group, consisting of 17 surgeons experienced in RIRS (>100 cases), was designated as Group S (Surgeons). Since Group S participants had already mastered theoretical knowledge, they did not attend the theoretical lecture sessions.

During the hands-on training, all participants were given a chance to try one out from start to finish under the supervision of the trainers. Afterward, the participants were allowed to go through the procedure from start to finish, again under the supervision of the trainers. *Flex-X2 Fiberscope Ureteroscope (Karl Storz)* and *NGage® Nitinol Stone Extractor (Cook Medical)* were used during the training on the 3D-AUSM station. At the VRRS station, the model was experienced by the participants as an application run on the computer.

The structured training steps determined for both models were as follows: (1) ability to exposure the urethra, (2) ability to exposure right ureteral orifice, (3) ability to exposure complete ureter, (4) ability to exposure intrarenal collecting system, (5) ability to relocate stone from upper to lower calyx, (6) ability to keep scope centered and avoid excessive trauma.

**Session IV – Collection of surveys and data forms:** Before the training, mentors were provided with a standard checklist and evaluated each step using a Likert scale from 0 to 10, where 0 indicated “complete failure” and 10 indicated “perfect success”. The total skill score for each participant was calculated by summing the scores of the individual steps. Additionally, the time taken for the first five steps was recorded. After completing the training, participants filled out a survey form evaluating the content, face, and construct validity of the models, using a Likert scale from 0 to 5.

### 2.3. Statistical analyses

Normality of the data was assessed using the Shapiro-Wilk test, as the number of participants in the groups was below 50. To compare the data obtained from Group T and Group S on the 3D-AUSM and VRRS, the Paired Sample T-test was used for normally distributed data, and the Wilcoxon Signed-Rank test was applied for nonnormally distributed data. Comparative results between Groups T and S were obtained using the Independent Samples T-Test for normally distributed data, and the Mann-Whitney U test for non-normally distributed data. Statistical analyses were performed using SPSS 20 (IBM, USA).

## 3. Results

All trainees participated in hands-on workshops with both the 3D-AUSM and VRRS. Mentors recorded the time spent during each training phase and evaluated participants’ skills on a 0–10 Likert scale for each structured step.

### 3.1. Analysis of elapsed times

The times recorded by mentors for each group while performing structured steps with both models are shown in seconds, as presented in Table 1. Group S completed the procedures significantly faster than Group T in almost all steps for both models. The most time-consuming steps for both groups were “exposing the intrarenal collecting system” and “relocating the stone”. Additionally, the total time spent on the 3D-AUSM was significantly higher than on the VRRS for both groups (Group T: p = 0.001, Group S: p < 0.01).

### 3.2. Analysis of skill scores

Mentors assigned skill scores (0–10 Likert scale) for each group during the structured steps using both models, as shown in Table 2. Group S scored significantly higher in all steps and total skill scores in both VRRS and 3D-AUSM compared to Group T (p < 0.001). No significant difference was found in the total skill scores between the two models for Group S (Total Skill Score: p = 0.269). However, in Group S, the “ability to keep scope centered and avoid excessive trauma” step received lower scores with 3D-AUSM (p = 0.027). Group T performed significantly better in all steps and total skill scores with VRRS than with 3D-AUSM (p < 0.001). The lowest skill scores for Group T in both models were in the “ability to keep scope centered and avoid excessive trauma” step (3D-AUSM mean: 3.0, VRRS mean: 4.16).

### 3.3. Content, face, and construct validation

After training, participants completed a survey evaluating the modalities using a Likert scale (0–5) for content, face, and construct validation, as presented in Table 3. For content validation, participants rated whether the models covered key steps of the RIRS procedure and aligned with current surgical techniques. Both groups gave high scores, indicating that the models fulfilled these criteria, with no significant difference between the two modalities (p > 0.05). Participants evaluated realism, training environment, and overall satisfaction for face validation. While all participants rated the models positively, Group S rated VRRS significantly lower for “overall satisfaction” than 3D-AUSM (p = 0.021), whereas Group T rated both similarly. For construct validation, questions focused on applying theoretical knowledge in hands-on training, skill development, and competence in RIRS, which were only asked to Group T. Group T gave high scores for both models, indicating improved skills and confidence posttraining, with no significant difference between the two models.

## 4. Discussion

RIRS is a widely used and effective method for treating kidney stones, offering high success rates and low complication risks, particularly in experienced hands [11]. However, like other endourological interventions, RIRS differs from traditional open surgery due to the lack of tactile feedback, which poses a key challenge in the training process [12]. While experienced surgeons achieve high success with low complication rates, novice surgeons at the early stages of their learning curve may encounter significant complications [3]. These complications range from superficial ureteral injuries, which can lead to strictures, to severe injuries such as ureteral avulsions that may necessitate renal autotransplantation [13]. Gaining initial experience through training methods outside the operating room can significantly reduce the occurrence of these complications.

These challenges have emphasized the need for alternative methods to effectively train surgeons in RIRS [14, 15]. Traditional surgical training on live patients in the operating theater can be supplemented by alternatives such as animal models, cadaveric training, 3D-printed models developed using 3D modeling technology, and VR/AR (virtual reality/augmented reality) applications [7, 16]. However, animal models have notable drawbacks, including high costs, the risk of biological contamination, anatomical differences from human systems, and ethical concerns due to the need to sacrifice animals [17, 18]. Similarly, cadaveric training faces challenges such as the degradation of tissue properties post-mortem, risks of contamination, and medicolegal and ethical issues related to cadaver supply [16]. In light of these limitations, we aimed to develop two new training methods using 3D modeling technologies to provide continuous and effective RIRS training without compromising patient safety or encountering biological contamination and medicolegal risks.

In this study comparing two different training models, when comparing the time spent by the groups while using the models, it was an expected finding that experienced surgeons (Group S) in both the 3D-AUSM and VRRS models completed the procedures in a shorter time than Group T due to their previous familiarity with the RIRS method. In both models, the most difficult parts of the structured steps were the most time-consuming steps, “exposure of the intrarenal collecting system”, and “relocation of the stone from the upper calyx to the lower calyx”. Unlike the steps with a single simple goal, such as exposure of the urethra, exposure of the correct ureteral orifice, and exposure of the complete ureter, these two steps had to be accomplished in a single complex step using different skills.

When comparing the time spent using each model, it is noteworthy that both groups completed the procedures faster in the VRRS model than in the 3D-AUSM. We believe this significant difference is due to the need for a real fURS device in the 3D-AUSM model. This requirement gave experienced surgeons an advantage, not only in time efficiency but also in the skill scores provided by the mentors. In contrast, since no real device was required for the VRRS model, and the training occurred entirely in a computer-simulated environment, the success scores of the inexperienced group were significantly higher compared to the 3D-AUSM.

When examining the skill scores, both groups received lower scores in the “keep the scope centered and avoid excessive trauma” step. The difficulties faced by the inexperienced group can be attributed to their limited exposure to basic endourological skills. However, the experienced surgeons’ challenges in this step may have different explanations. In actual patients, the RIRS procedure takes place in a fluid environment made up of a mixture of urine and physiological serum, administered through the URS canal. Unlike the rigid structure of a 3D-printed model, the real collecting system is highly flexible. The irrigation fluid helps expand the ureteral lumen, allowing more room for the scope to move. Additionally, this fluid medium makes the scope more slippery, facilitating easier movement. These factors combined may explain the lower skill scores for experienced surgeons in this particular step.

Both models received high scores from participants in the evaluations of content, face, and construct validation. Participants noted that they found both models to be realistic and satisfactory, that the training covered all essential aspects of the current RIRS procedure, and that the training made them feel more competent. Feedback from participants in similar studies also reported high ratings, demonstrating that computer-assisted 3D training modalities are well-received by users [9, 10]. Although inexperienced participants found both models equally satisfactory in terms of face validation, experienced surgeons gave lower scores for the VRSS. This may be because surgeons with real surgical experience felt that virtual reality simulations do not fully replicate the complexity of real-life procedures. In both modalities, the fact that the experienced surgeon group completed the procedures more quickly and achieved higher success scores indicates that real-world surgical experience plays a crucial role in model usage. This finding also supports the argument that these models are validated in replicating real surgical procedures.

Although these training modalities cannot entirely replace surgical experience with actual patients, they play a crucial role in acquiring fundamental surgical skills [3]. When comparing the two modalities, it is clear that 3D-printed models stand out for their low costs and realistic anatomical features, while VR simulators provide the benefit of unlimited repetition and the ability to train in a low-stress environment [19]. These modalities offer a safer alternative by avoiding the ethical and medicolegal risks associated with procedures performed by inexperienced surgeons during traditional patient-based training. Moreover, through the structured and rational use of these tools, standardized education can be achieved globally using predetermined educational steps. The ability to use 3D-printed models and VR simulators-produced at a one-time cost-without incurring additional expenses highlights a significant advantage of 3D technologies over other training models.

Recent technological advancements suggest that 3D models will play an increasingly important role in the future of medical education. This is especially true in fields like Urology, where endoscopic procedures are becoming more prominent due to ongoing technological developments. Training modalities that allow for the acquisition of essential surgical skills without the risk of patient harm are becoming indispensable. We believe that the results of this study will significantly contribute to the standardization and effective implementation of RIRS training. These models will undoubtedly facilitate standardized training at a low cost, ensuring the consistent acquisition of fundamental skills.

## 5. Limitations

While this study provides valuable insights into the effectiveness of 3D-printed anatomical models and VR simulators for RIRS training, several limitations must be acknowledged. One of the most significant limitations is the absence of laser fragmentation simulation in both models. Laser lithotripsy is a critical step in RIRS, and improper technique can lead to complications such as mucosal injury, perforation, and excessive stone migration. The current training modalities primarily focus on ureteroscope handling, anatomical orientation, and stone relocation; however, they do not provide a realistic environment for laser energy application. Future developments should aim to integrate laser fragmentation simulations, either through augmented reality (AR)-assisted feedback systems or physical models incorporating laser-compatible materials to better mimic real-life scenarios.

Additionally, although various surgical skills were evaluated in this study, all experienced surgeons would agree that surgical competence extends beyond a collection of technical skills. Factors such as communication in the operating room, the ability to make decisions in challenging scenarios and under stress, and leadership play a crucial role in a successful surgical process [20]. This limitation could be addressed by incorporating different scenarios into the structured training steps of the developed modalities.

Another limitation is the short-term nature of this study. It did not evaluate the long-term sustainability of these skills or the lasting impact of the training. Future studies should explore the long-term effects of these models to gain a deeper understanding of the durability of the training outcomes.

## 6. Conclusions

In conclusion, the use of 3D-printed models and VR simulators offers a safe, effective, and cost-efficient method for developing fundamental surgical skills. These tools not only provide the opportunity to practice in a risk-free environment but also allow for the standardization of training across various institutions. Their ability to offer repeatable and customizable learning experiences makes them increasingly important in modern surgical education.

## Figures and Tables

**Table 1:** Times recorded by the mentor in seconds while participants completed structured steps in 3D-AUSM and VRRS (experienced surgeons: Group S, trainees: Group T). Group S completed the steps significantly faster than Group T. Total time was higher for 3D-AUSM than for VRRS in both groups (Group T: p=0.001, Group S: p<0.01). (3D-AUSM: 3D-Printed Anatomical Urinary System Model,

	Group T (n=32)						Group S (n=17)						*p*			
	3D-AUSM (α)			VRRS (β)			3D-AUSM (γ)			VRRS (δ)			*(α, β, γ, δ)*			
Time of the Steps (Second)	Mean	Median	25–75	Mean	Median	25–75	Mean	Median	25–75	Mean	Median	25–75	*α vs β*	γ vs δ	*α vs γ*	*β vs δ*
The urethral exposure time	42.69	43.50	31.25–52.75	35.25	36.00	28.25–41.75	10.59	10.00	8.00–13	6.35	6.00	5.00–7.00	0.025^a^	<0.001^a^	<0.001^d^	<0.001^d^
Correct ureteral orifice exposure time	61.19	56.00	40.50–80	62.09	60.50	50–72.75	11.82	11.00	9.00–14	9.24	8.00	7.00–11.00	0.883^a^	0.036^b^	<0.001^c^	<0.001^c^
Complete ureteral exposure time	84.31	81.00	67.50–104	67.78	70.00	56.25–81.75	21.47	20.00	17.50–25.50	15.12	15.00	11.00–17.50	0.018^a^	0.006^a^	<0.001^d^	<0.050^d^
Intrarenal collecting system exposure time	110.50	112.50	86.50–127.5	88.28	83.50	58–102.75	44.18	43.00	34.50–57	28.35	28.00	23.00–31.50	0.024^b^	0.001^a^	<0.050^d^	<0.001^c^
Stone relocate time	155.47	149.50	122.50–178.25	119.69	123.00	88.50–143.5	48.59	46.00	34.50–62.50	38.29	36.00	28.50–49.50	0.002^b^	0.003^a^	<0.001^c^	<0.001^d^
Total procedure time	454.16	436.50	378.50–523	373.09	371.50	323.75–415.75	136.65	132.00	117.50–150	95.76	95.00	83.50–104.00	0.001^a^	<0.001^a^	<0.001^d^	<0.001^d^

VRRS: Virtual Reality RIRS Simulator, RIRS: Retrograde Intrarenal Surgery)

a Paired Sample T-test,

b Wilcoxon Signed-Rank Test,

c Mann-Whitney Test,

d Independent Samples T-Test.

**Table 2:** Skill scores (0–10) given by mentors for structured steps in 3D-AUSM and VRRS for experienced surgeons (Group S) and trainees (Group T). Group S consistently received higher scores than Group T across all steps and total scores for both models (p<0.001). Group T scored significantly higher on VRRS than on 3D-AUSM in total and individual steps (p<0.001). (3D-AUSM: 3D-Printed Anatomical Urinary System Model, VRRS: Virtual Reality RIRS Simulator, RIRS: Retrograde Intrarenal Surgery)

	Group T (n=32)						Group S (n=17)						*p*			
	3D-AUSM (α)			VRRS (β)			3D-AUSM (γ)			VRRS (*δ*)			*(α, β, γ, δ)*			
Skill Steps	Mean	Median	Min–Max	Mean	Median	Min–Max	Mean	Median	Min–Max	Mean	Median	Min–Max	*α vs β*	γ vs δ	*α vs γ*	*β vs δ*
Ability to exposure the urethra	4.81	4	3–7	5.94	6	4–8	9.29	9	8–10	9.12	9	8–10	0.002^b^	0.564^b^	<0.001^c^	<0.001^c^
Ability to exposure right ureteral orifice	4.09	4	2–6	5.47	6	3–7	8.47	9	7–10	8.76	9	7–10	0.002^b^	0.521^b^	<0.001^c^	<0.001^c^
Ability to exposure complete ureter	5.06	5	3–7	6.31	6.5	4–8	8.94	9	8–10	9.00	9	8–10	0.002^b^	0.808^b^	<0.001^c^	<0.001^c^
Ability to exposure intrarenal collecting system	4.56	5	2–7	5.75	6	3–8	7.94	8	6–10	7.88	8	7–10	0.03^b^	0.823^b^	<0.001^c^	<0.001^c^
Ability to relocate stone from upper to lower calyx	4.53	4	3–6	5.75	6	4–7	8.65	9	6–10	8.29	8	7–10	0.001^b^	0.425^b^	<0.001^c^	<0.001^c^
Ability to keep scope centered and avoid excessive trauma	3.00	3	1–6	4.16	4	2–7	7.76	7	6–10	8.82	9	7–10	0.012^b^	0.027^b^	<0.001^c^	<0.001^c^
Total skill score	26.06	26	21–32	33.38	33	26–43	51.06	51	48–55	51.88	52	48–56	<0.001^a^	0.269^b^	<0.001^d^	<0.001^d^

a Paired Sample T-test,

b Wilcoxon Signed-Rank Test,

c Mann-Whitney Test,

d Independent Samples T-Test.

**Table 3:** Survey scores (0–5) from content, face, and construct validation for 3D-AUSM and VRRS. Both groups gave high scores, indicating that the models cover essential RIRS steps and align with surgical techniques (content validation). Group S rated overall satisfaction for VRRS lower than for 3D-AUSM (p=0.021), while Group T rated both models similarly in terms of realism and training effectiveness (face and construct validation). There were no significant differences between models in content validation scores (p>0.05). (3D-AUSM: 3D-Printed Anatomical Urinary System Model, VRRS: Virtual Reality RIRS Simulator, RIRS: Retrograde Intrarenal Surgery)

	Grup T (n=32)							Grup S (n=17)							
	3D-Printed			VR Model			*p*	3D-Printed			VR Model			*p*	
	Mean	Median	Min–Max	Mean	Median	Min–Max		Mean	Median	Min–Max	Mean	Median	Min–Max		
**Content Validation**	1. Does the model cover important aspects of the RIRS procedure?	4	4	3–5	3.66	4	2–5	0.11^a^	4	4	3–5	3.65	4	2–5	0.19^a^
	2. How compatible is the model with current surgical techniques and protocols?	4.03	4	3–5	3.69	4	2–5	0.106^a^	4.12	4	3–5	4.35	4	3–5	0.285^a^
**Face Validation**	1. What do you think about the realism and satisfaction of the model?	3.97	4	3–5	3.66	4	2–5	0.062^a^	3.94	4	3–5	3.41	3	2–5	0.08^a^
	2. How would you evaluate the suitability of the physical environment used during the training?	4.06	4	3–5	3.72	4	2–5	0.07^a^	4	4	3–5	4.06	4	3–5	0.796^a^
	3. How would you rate this training in terms of your overall satisfaction?	3.97	4	3–5	3.88	4	2–5	0.554^a^	4.18	4	4–5	3.71	4	3–5	0.021^a^
Construct* **Validation**	1.How comfortable did you feel applying what you learned in theoretical training during hands-on training?	4	4	3–5	3.94	4	2–5	0.723^a^	*	*	*	*	*	*	-
	2.How much did working on the model during hands-on training improve your surgical skills?	4	4	3–5	3.84	4	2–5	0.362^a^	*	*	*	*	*	*	-
	3.Do you feel more competent to perform RIRS procedures after hands-on training?	3.97	4	3–5	4.13	4	3–5	0.322^a^	*	*	*	*	*	*	-

* Construct validation questions were addressed only to Group T.,

a Wilcoxon Signed-Rank Test.
